# A Syndrome Affecting All Five Sense Organs: A Rare Congenital Disorder of Kabuki Makeup Syndrome With Multiple Pre-auricular Skin Tags

**DOI:** 10.7759/cureus.69455

**Published:** 2024-09-15

**Authors:** Navin Umapathy, Vaanmathi Azhagar Nambi Santhi, Balakrishnan T, Lal D.V Nair

**Affiliations:** 1 Pediatrics, Saveetha Medical College and Hospitals, Saveetha Institute of Technical and Medical Sciences, Saveetha University, Chennai, IND

**Keywords:** craniofacial dysmorphism, kabuki syndrome(ks), kmt2d gene, pediatric development, whole-exome sequencing

## Abstract

Kabuki syndrome is an autosomal dominant disorder characterized by distinct facial features, including long palpebral fissures, a short columella with a flat, broad nasal tip, ptosis, and cleft lip/palate. The syndrome was named for the resemblance of the facial features to the make-up worn by traditional Kabuki performers. We report the case of a 10-month-old female infant admitted for cleft palate repair. The patient exhibited normal developmental milestones but had recurrent respiratory infections secondary to her cleft lip and palate. The child presented with significant facial dysmorphism, including long palpebral fissures with ptosis, multiple preauricular skin tags, a short columella with a depressed nasal tip, and microtia. These findings prompted differential diagnoses of Goldenhar syndrome, branchio-oculo-facial syndrome, and Kabuki syndrome. Whole exome sequencing confirmed a diagnosis of Kabuki syndrome. Given the autosomal dominant nature of this disorder, early identification and management of potential complications are crucial, as is parental counseling regarding the implications for future pregnancies.

## Introduction

Kabuki syndrome is a rare congenital disorder with atypical facial features, such as long palpebral fissures with eversion of the lateral third of the lower eyelid and short columella with a depressed nasal tip [1,2]. According to medical literature, around 400 genetically proven cases of Kabuki syndrome have been reported worldwide. Most children with Kabuki syndrome can lead average lives with supportive care. There are many phenotypic variants of Kabuki syndrome.

It was first reported by Japanese physicians in 1981 in a child with facial features resembling the style of Japanese Kabuki makeup artists. Hence, it was initially known as “Kabuki makeup syndrome” due to its resemblance to stage makeup in traditional Kabuki theatre [2,3]. Kabuki syndrome can be diagnosed genetically by identifying mutations in genes KMT2D and KDM6A, although many mutation variants exist. According to the diagnostic consensus, a child should have mutations in the KMT2D/KDM6A gene variants along with two or more typical clinical features, from arched and broad eyebrows, short columella with a depressed nasal tip, large prominent ears, and persistent fingertip pads [1,4].

Kabuki syndrome has an estimated prevalence of one in 32,000 live births. It poses diagnostic challenges and often requires a multidisciplinary approach to management [3,5]. This case report aims to detail the clinical presentation, diagnostic process, and management strategies of a patient diagnosed with Kabuki syndrome, providing insight into the complexities of this multifaceted condition and contributing to the growing body of knowledge surrounding its clinical spectrum and genetic underpinnings.

Certain congenital heart defects, such as atrial septal defect, ventricular septal defect, hypoplastic left heart syndrome, coarctation of the aorta, and mitral stenosis, are commonly noted among children with Kabuki syndrome [4,6]. Many affected children also exhibit a developmental delay, which is commonly seen after their first year of life [3,7].

Dental malformations, such as irregular dentition, malformed jawline, cleft lip and palate, and tongue muscle wasting, are seen in few cases of Kabuki syndrome [8,9]. Hearing loss, hip dislocation, seizures, and scoliosis are some atypical presentations reported in children with Kabuki syndrome [10,11]. As such, this syndrome has a variety of clinical features and newer variants in mutations that have been reported in medical literature.

## Case presentation

**Figure 1:**
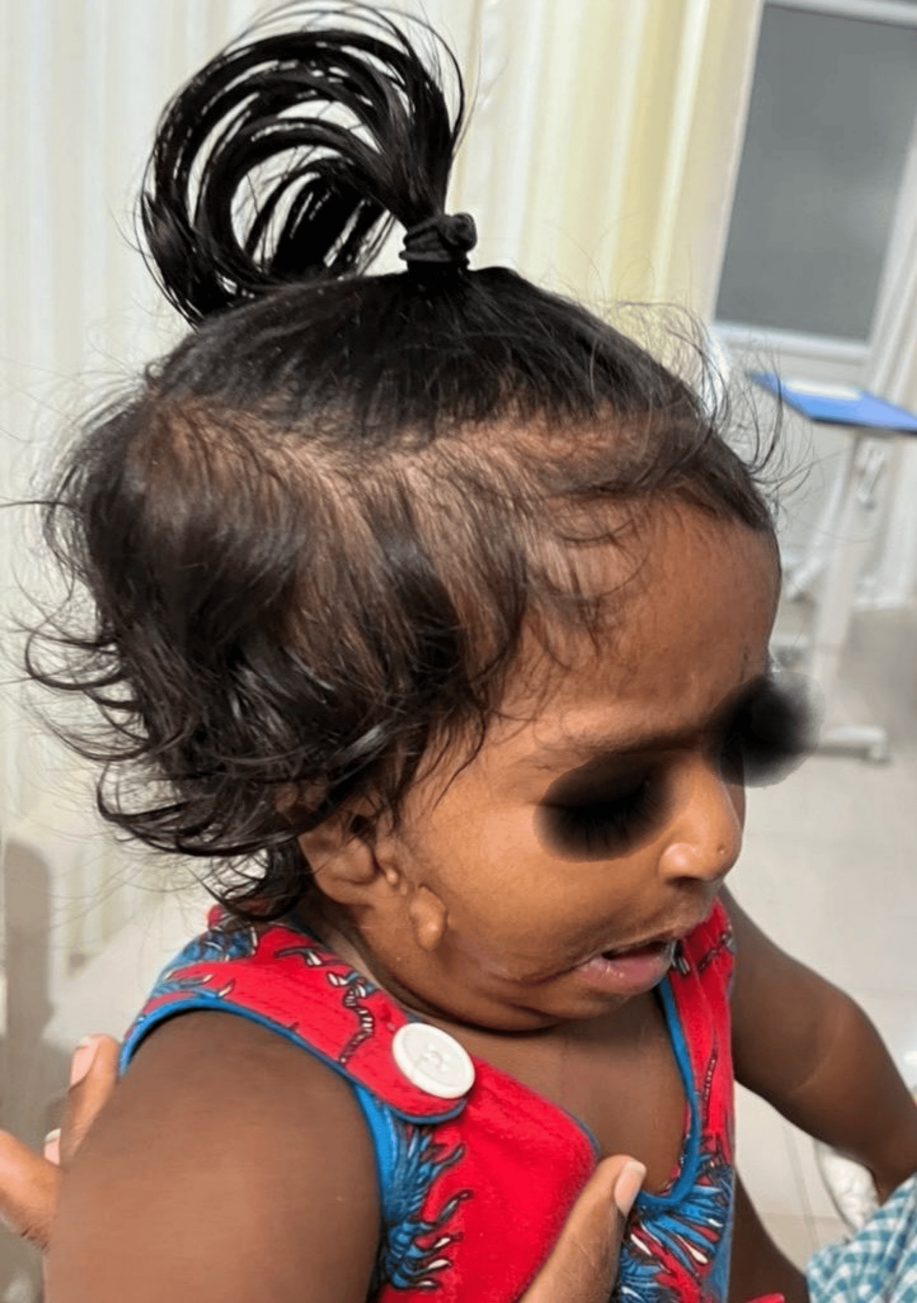
Multiple pre-auricular skin tags with right ear microtia

**Figure 2:**
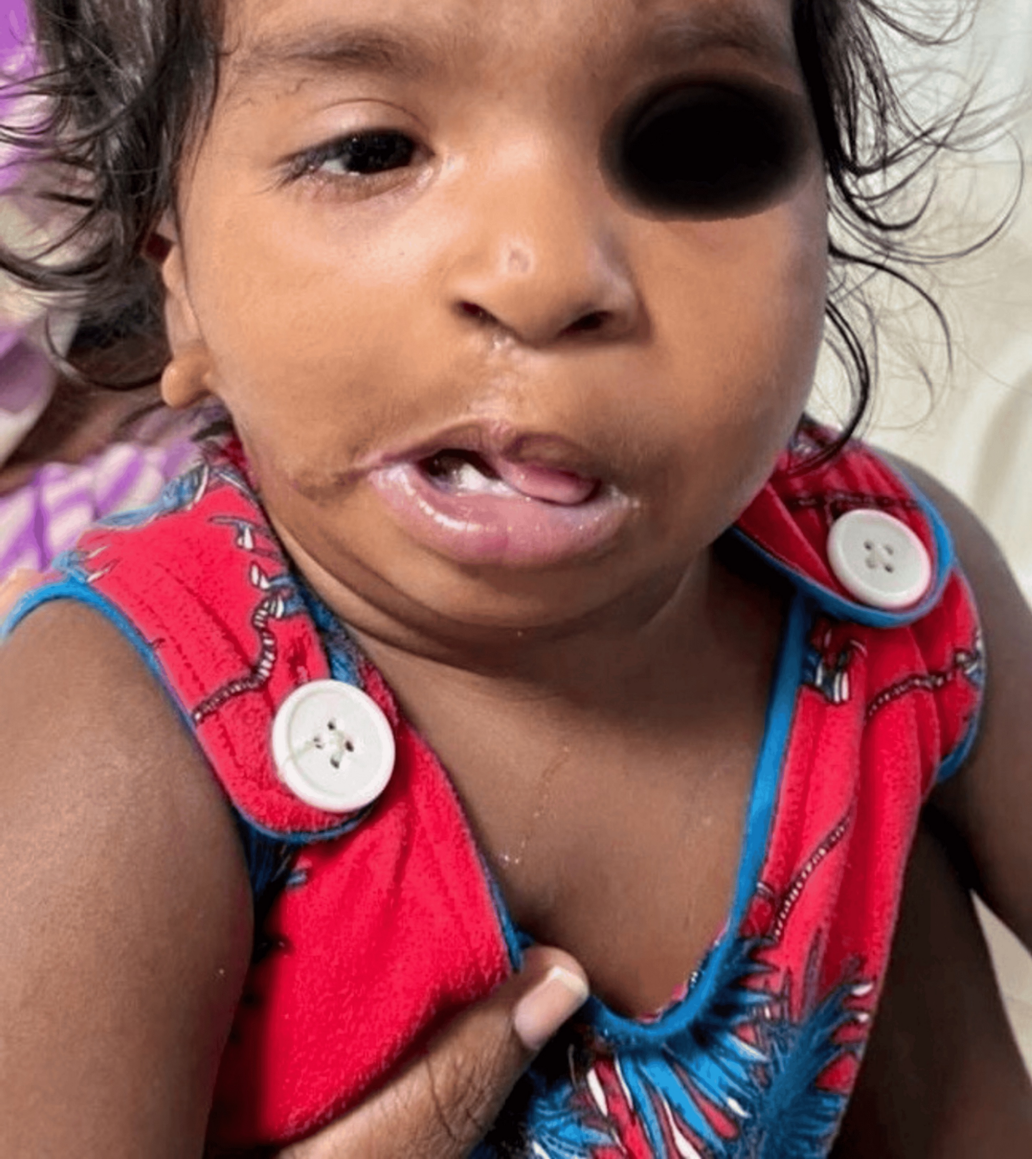
Dysmorphic facies with right eye ptosis and cleft lip operated

A 10-month-old female toddler was brought to our outpatient department with a cleft lip and palate and was admitted for surgical correction. On examination, she was noted to have dysmorphic facial features, including a broad eyebrow on the right lateral eyelid, multiple pre-auricular tags on the right cheek, right complete cleft lip and palate, partial ptosis of the right eye, blepharophimosis, right ear microtia, mild scoliosis, and hemifacial microsomia with incomplete Tessier number 7 lateral facial cleft (Figure 1). The child is the firstborn to second-degree consanguineous parents; she was delivered vaginally at term with no postnatal complications and was dysmorphic at birth (Figure 2).

The mother did not have regular antenatal visits, and an anomaly scan was not done. After birth, the child was breastfed exclusively for six months; a history of occasional aspiration was noted, and the child was developmentally normal for her age (10 months). She was immunized as per the national immunization schedule. The initial surgery to correct the cleft lip occurred at four months of age. The current admission was for palatoplasty. Basic blood investigations were done for pre-operative workup, which showed anemia of infancy with mild leukocytosis and positive C-reactive protein. Meanwhile, the child had a cough and fever, so blood cultures were sent and reported to be negative, and she was symptomatically treated. The coagulation profile and serum procalcitonin were within normal limits. Coronavirus disease 2019 (COVID-19) reverse transcription polymerase chain reaction (RT-PCR) was negative. An abdominal ultrasound and chest radiograph were done, and no abnormality was detected. An echocardiogram was done to evaluate potential congenital disabilities and was found to be normal. An ophthalmologist’s opinion was sought for ptosis, with a recommendation to follow up when the child was two years old, stating that no intervention was required at present. The ocular fundus examination was reported as normal. A hearing assessment (brainstem-evoked response audiometry) noted mild hearing loss in the right ear and a normal range in the left ear. First, Goldenhar syndrome was considered, which can have ocular and auricular malformations, but mandibular hypoplasia and vertebral anomalies were not evident. Features such as skin tags and auricular and eye defects favored branchio-oculo-facial syndrome [2,3]. However, the presence of these features meant we also had to consider Kabuki syndrome. In order to confirm the diagnosis, we undertook whole exome sequencing, which confirmed a rare variant (KMT2D deletion on exon 32, c.6578C variant, heterozygous with autosomal dominant inheritance) of Kabuki syndrome. Corrective plastic surgery addressed the cleft palate and lateral cleft, and the postoperative period was uneventful.

## Discussion

Kabuki syndrome is a rare disorder involving defects in all five sense organs, including the nose, ears, eyes, lips, and oral cavity, along with skin tags. Since it is an autosomal-dominant disorder, it is easily inherited through generations with more variant phenotypes. Children with Kabuki syndrome usually have varying degrees of intellectual disabilities, developmental delays, and short postnatal statures, mostly evident by one year of age [3]. Early identification is beneficial in regard to the early anticipation of complications and management of the same. Exome sequencing assists parents in understanding any concerns for the child and planning for potential subsequent pregnancies. Tessier 7 lateral clefts are more commonly associated with hemifacial microsomia, whereas number 8 clefts are more associated with Goldenhar syndrome, though delayed psychomotor development helps in their differentiation. Though 50% of Kabuki syndrome patients have a cleft palate and congenital heart defects, lateral facial cleft, together with palatal cleft and the absence of congenital heart defects, makes this case an unusual presentation [4]. This genetic variant of Kabuki syndrome has not been reported to date. A detailed search of the Online Mendelian Inheritance in Man (OMIM) database revealed Kabuki-1 (OMIM#147920) with similar clinical features and a heterozygous mutation in the KMT2D gene. However, this variation stipulated an intellectual disability and postnatal short stature, which were not present in this child. Most cases of Kabuki syndrome result from spontaneous mutations. Therefore, parents can be counseled about this when considering the possibility of having a second child, with the index child being autosomal dominant. Few cases of X-linked dominance have been reported with mutations, mainly at KDM6A. Both KMT2D and KDM6A are considered part of a new class of “Mendelian disorders of epigenetic machinery” that affect the epigenetic machinery of expression of genes through histones [4]. KMT2D mutations, as in the index child, will cause altered expression of histones, which cause the DNA to be kept in an “open” configuration to be read for the gene to express. KDM6A removes the marks that keep the DNA closed so that the DNA can be opened and read [5].

Since development of the index child is normal so far without any evidence of short stature, further developmental follow-up may reveal milder forms of intellectual disability at a later date [6]. If the above parameters are normal at follow-up, then this variant may turn out to be the first reported case with normal development and stature among the reported Kabuki syndromes.

## Conclusions

Parents were counseled regarding the child’s condition and inheritance risk. Effective speech interventions were delivered after corrective surgery and complete developmental assessment. The absence of seizures, intellectual disability, and congenital heart defects, along with near-normal hearing, ensures a good prognosis for this variant. A clinical examination helps to identify closer differentials to determine the genetic workup. Though grossly dysmorphic, Kabuki syndrome can be differentiated from its close differentials by clinical examination itself. However, atypical presentations make it difficult, necessitating genetic analysis to prognosticate, as certain Kabuki variants will allow for better cognition and normal development. Exome sequencing also helps counsel the parents on inheritance patterns and future pregnancies. This rare case indicates that many genetic syndromes can have atypical presentations due to emerging new gene mutations; therefore, exome sequencing plays a vital role in diagnosis and anticipation.
